# Preparation and Anodizing of SiCp/Al Composites with Relatively High Fraction of SiCp

**DOI:** 10.1155/2018/8945729

**Published:** 2018-02-26

**Authors:** Bin Wang, Shengguan Qu, Xiaoqiang Li

**Affiliations:** ^1^Guangdong Key Laboratory for Processing and Forming of Advanced Metallic Materials, South China University of Technology, Guangzhou 510640, China; ^2^National Engineering Research Center of Near-Net-Shape Forming for Metallic Materials, South China University of Technology, Guangzhou 510640, China

## Abstract

By properly proportioned SiC particles with different sizes and using squeeze infiltration process, SiCp/Al composites with high volume fraction of SiC content (Vp = 60.0%, 61.2%, 63.5%, 67.4%, and 68.0%) were achieved for optical application. The flexural strength of the prepared SiC_p_/Al composites was higher than 483 MPa and the elastic modulus was increased from 174.2 to 206.2 GPa. With an increase in SiC volume fraction, the flexural strength and Poisson's ratio decreased with the increase in elastic modulus. After the anodic oxidation treatment, an oxidation film with porous structure was prepared on the surface of the composite and the oxidation film was uniformly distributed. The anodic oxide growth rate of composite decreased with SiC content increased and linearly increased with anodizing time.

## 1. Introduction

The aluminum matrix composites reinforced by SiCp particles have been extensively applied in industry for their low density and high specific strength. In recent years, these materials with high fraction of SiC_p_ particle (>60%) are used in space mirror, owing to their excellent thermal conductivity and low coefficient of thermal expansion (CTE) [1]. SiCp/Al composite with relatively high SiC content are selected as structural substrates for a space mirrors [2]. Guo et al. [3] analyzed that the closer the glass and matrix of thermal expansion coefficients between them, the better the combination. Zhang et al. [4] prepared a plane mirror with an open back lightweight structure with SiCp/Al composite with a light advantage. However, due to high volume fraction SiCp/Al with high hardness and low plasticity, it is difficult to machine this kind of material to achieve high surface microroughness; otherwise it cannot reach the optical function [5]. At present, the glass coating technique, which has been taken as an important modification technique to bond the glass on the SiCp/Al composite surface, has attracted more interest, because the higher polishability of the SiCp/Al composite surface can be obtained by using this technique [6, 7]. Compounding optical glass and composites improves the polishing properties of the composites [8]. In order to obtain a high profile and surface roughness, the mirror is polished by using the ultrasmooth polishing. Several researchers have attempted to overcome the bonding strength between metal and glass. Chanmuang et al. [9] prepared the borosilicate glass-to-Kovar joint by bonding glass to the alloy, and the joint has a bonding strength of 4.3 MPa. Joining was preformed by fusion of the glass, which wetted the alloy, at 1000°C after 15 min in the electric furnace. In traditional process, compounding of composite and glass is operated under high temperature. If we adopt the anodic oxidation technology, the binding temperature can be reduced. It is significant to save energy.

The bonding strength of SiCp/Al composite-glass components plays an important role in the reliability. The bonding property at glass/metal interface is deemed as the key benchmark for assessing the quality of the space mirror, because it will be posited in the harsh serving condition. Mantel [10] indicated the initially prepared oxidation film at the glass/metal interface was favorable to enhance the bonding property.

As far as Al matrix composite, it is well known that the thickness of alumina film under natural condition is only several nanometers, which is difficult to form a stable metal/glass bonding interface. After the anodizing process on the SiC_p_/Al substrate, an oxidation film with thick enough dimension on the surface of aluminum could be obtained. Aluminum is a potential candidate metal for forming an oxidation film with substantial thickness on its surface. However, the aluminum layer on the exposed surface of Al ingot is too thin to satisfy the demand of promoting the bonding property at glass/metal interface. In the past decades, techniques such as anodizing and microarc oxidation were proposed for anticorrosion of Al substrate. Anodizing is an effective and commercial method for obtaining a thick oxidation film on Al surface, which is also suitable for the industrial mass production.

So far, there have been few reports about anodized SiCp/Al composite, even less high volume fraction SiCp/Al composite. Because the local melt structure around SiC particle affects the microstructures of the composites and the properties of the interface [11], compounding temperature must be lower than the solution temperature of the matrix. However, researchers have paid little attention to the melting point of anodized high volume fraction SiCp/Al composites. In order to obtain a perfect combination of composite and glass, the effect of the anodizing process on the oxide growth rates of composites and the solution temperature of composites needs to be investigated. For the aerospace application, combination of glass and alloy was often achieved at relatively high temperature 1000°C after 15 min in the electric furnace. In our previous work [12], the glass was combined to the SiCp/Al composite matrix with high content SiC particles with 45 *μ*m, 8 *μ*m, and 2 *μ*m addition by preparing an alumina layer on the surface of SiCp/Al composite in the electric furnace. Selecting an optical grade SiCp/Al composite with high SiC content as a space mirror material is propitious to promote the integration of the mirror and its supporting structure. However, till date, few reports are concerned with combining the glass on the Al matrix with high addition of SiC particles with vacuum hot-pressing compound. As a novel structural material for aerospace, composites have to possess good mechanical properties when the aerospace parts are in the service conditions of rapid cooling and heating, such as with high dimensional stability, high elastic modulus, and low Poisson's ratio.

## 2. Experiment

### 2.1. Materials and Procedures

The chemical compositions of the Al matrix are listed in Table 1. The green *α*-SiC (6H) powder with a purity of 99.9% was used in this experiment. In order to obtain the SiCp/Al composites with high SiCp fractions, the particles with the median diameters of 45 *μ*m, 8 *μ*m, and 2 *μ*m were ball-milled for 10 h and then homogenously mixed at room temperature. The size distribution, tested by Malvern laser particle size analyzer, of the particles after ball milling process was given in Table 2.

A hydraulic machine was carried out for the preparation of SiCp preform. In this process the mixed SiCp powder was compacted in a cylindrical graphite mold under a pressure of 25 MPa. Thereafter, the preform was sintered at 1600°C for 2 h and cooled down to room temperature in the atmosphere.

The SiCp/Al composite was manufactured by using the squeeze casting machine. The prepared preform was preheated to 700°C in a steel mold. The molten Al (superheated to 820°C) was infiltrated to the steel mold by applying the hydraulic pressure from 8 to 90 MPa. The steel mold was cooled down to the room temperature when the infiltration process was completed.

The formed SiCp/Al composites were machined into the standard tensile and flexural specimens according to GB/T 228.1-2010 and GB/T 232-2010, respectively.

Flexural samples were also fabricated according to the standard of GB/T 232-2010. The dimension for the flexural sample was 65 mm × 7 mm × 7 mm. The parallelepiped samples was machined with the size of 8 mm × 8 mm × 8 mm. The parallelepiped samples were used for anodizing process. Before anodizing operation, the samples was etched in KOH solution (50 g/L) at room temperature for 2 min. Thereafter, it was rinsed in distilled water. Chemical pickling process was carried out in terms of HNO_3_ solution (1 mol/L) at room temperature for 3 min. Again, the sample was cleaned by distilled water and dried in a drying oven. The anodizing electrolyte was sulfuric acid. The sulfuric acid concentration was 180 g/L; the polar distance was 3 cm; the anodic current density was 1.6 A. An aluminum alloy plate was used as the cathode materials. Sulfuric and nitric acids were analytical grade chemicals. Different anodic oxidation times were employed in order to obtain different thickness of the oxidation film. The time durations for the anodizing process were independently set at 5 min, for 10 min, for 15 min, for 20 min, for 25 min, and for 30 min. The parallelepiped samples were mechanically ground P 1500 grade paper and then polished.

### 2.2. Testing Methods and Characterization

**Figure 1:**
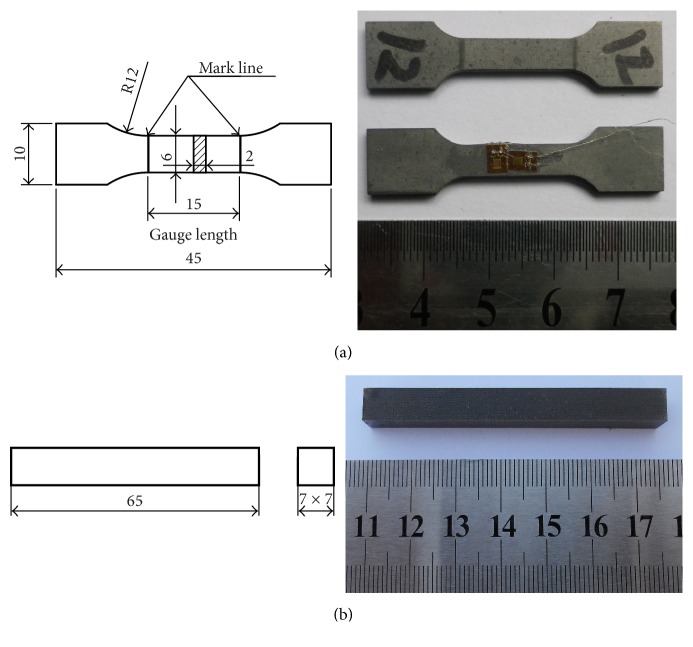
The schematic of tensile and flexural specimen size and the photo of test sample.

In order to obtain a better interface bonding strength, high volume fraction SiCp/Al was anodized prior to vacuum hot-pressing. Two sets of strain gauges that scatter in orthogonal planes were stuck on the tensile specimens. The gauge length was marked on the surface of the tensile specimens (Figure 1). The strains of the specimens were tested at room temperature on a MTS test machine. Based on strain measurements of the composites, Poisson's ratio of the composites was calculated. By measuring the change in the length of tensile sample and the cleavage fracture stress, the elastic modulus of the composites was calculated. The flexural strengths of the samples were tested at room temperature on a MTS test machine. The test method is a three-point bending test. The micromorphologies of the mixed SiC particles and the fracture surface of tensile samples and flexural samples were observed by a NOVA NANOSEM 430 scanning electron microscope (SEM).

X-ray diffraction (XRD) analysis of the parallelepiped samples was carried out on a SIEMENS D8 ADVANCE diffractometer using Cu radiation. Electron backscattered diffraction (EBSD) was used to evaluate the oxide layers of the samples. The oxygen content in the anodized samples was analyzed by Energy Dispersive Spectrometer (EDS). The parallelepiped samples were sputtered with platinum for 70 seconds to characterization. SEM was employed to observe the microstructure of the anodic film. The differential Scanning Calorimetry (DSC) measured the endothermic peak of the composites. The heating rate was 5°C/min. The DSC scanning was initiated at 30°C and completed at 750°C.

## 3. Results and Analysis

### 3.1. Mechanical Properties of Composites

#### 3.1.1. Flexural Strength

Testing results in Table 2 show that the flexural strengths of composites are 569, 554, 548, 544, and 483 MPa for the 60.0%, 61.2%, 63.5%, 67.4%, and 68.0% composites, respectively. The flexural strength of the matrix is 398 MPa (Table 3 for the properties of aluminum alloy), and the flexural strength of the SiC is 550 MPa. The flexural strength of the composites is greater than the flexural of the matrix; even the flexural strengths of 60% and 61.2% composites are higher than the flexural strength of SiC. This indicates remarkable enhancement in flexural strength of composites with an addition of SiC particles.

**Figure 2:**
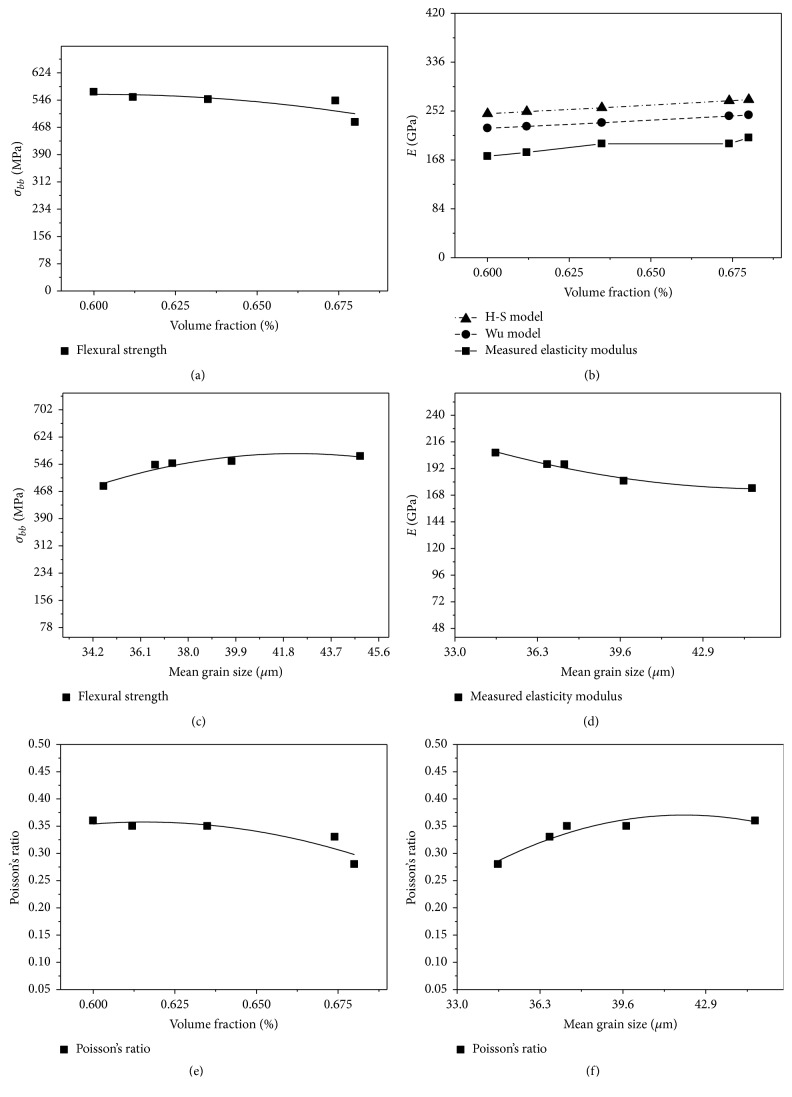
(a) Effect of SiC vol.% on flexural strength, (b) effect of SiC vol.% on elastic modulus, (c) effect of mean grain size on flexural strength, (d) effect of mean grain size on measured elasticity modulus, (e) effect of SiC vol.% on Poisson's ratio, and (f) effect of mean grain size on Poisson's ratio.

**Figure 3:**
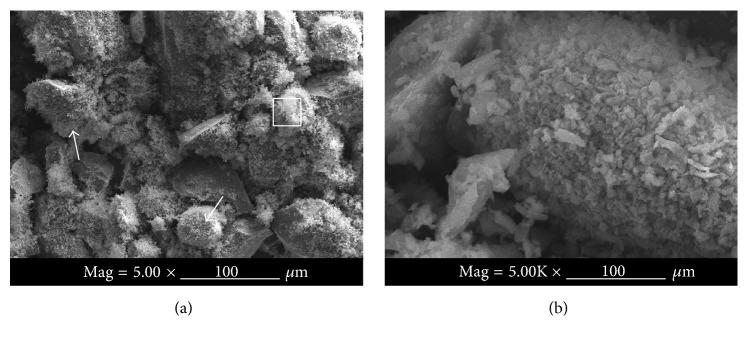
(a) SEM images of the mixture SiC particle (gradation composition and proportion 45 *μ*m : 8 *μ*m : 2 *μ*m = 1000 : 250 : 250) and (b) high magnification image of the framed area in (a).

The effect of SiC content to the flexural strength of composites is presented in Figure 2(a). It is observed in Figure 2(a) that the flexural strengths of composites have a clear tendency to decrease with the increase of SiC particle volume fraction; in particular the flexural strength produced at a rapid decrease in the value of SiC content from 67.4% to 68.0%. This can be ascribed to two reasons: on the one hand, the higher the volume fraction of SiC particle and the smaller the size of SiC particle the easier the agglomeration of SiC particles [13, 14]. In this experiment, composites are prepared with the mixture of SiC particles infiltration in molten aluminum. The mean grain sizes of SiC particle are 45 *μ*m, 8 *μ*m, and 2 *μ*m. The size of SiC particle mixture is refined after ball milling. It is clearly seen from Figure 3(a) (white arrow) that there are many fine particles in the mixture, and these fine particles cluster together. Some fine particles are very fine, with sizes reaching nanometers (Figure 3(b)). Figure 3(b) shows that the fine particles can form agglomeration easily. The agglomeration of fine particles prevents the infiltration of molten aluminum into a SiC preform and results in a degradation of the interface bonding performance between SiC and matrix. The stress transfer at the SiC-matrix interface becomes more inefficient with the increase of SiC content. So, the flexural strengths of composites decrease with increasing particle loading. Another reason is related to the brittleness of SiC. Composites will become more brittle with the increase of SiC particle content. The increase of brittleness causes a decline in the flexural strength of composites [15].

Testing data in Table 2 indicates that the mean grain sizes of SiC particles in composites are 44.87, 39.74, 37.38, 36.39, and 34.63 *μ*m for the 60.0%, 61.2%, 63.5%, 67.4%, and 68.0% composites, respectively. The effect of SiC particle size on the flexural strengths of composites is shown in Figure 2(c). As depicted in Figure 2(c), for smaller mean grain size of SiC particles (34.63–37.38 *μ*m), there is a prompt increase in the flexural strength with increasing particle sizes. For larger mean grain sizes of SiC particles (39.74–44.87 *μ*m), the increase in the flexural strength is no longer significant. The results imply that SiC particle size has an effect on SiC-matrix interface adhesion. The mean grain size of SiC particles is related to the content of 2 *μ*m particles. With the content of 2 *μ*m particle decreasing, the content of fine particles decreases, and the agglomeration of SiC particles reduces. Desirable infiltration results can be obtained during squeezing molten aluminum into SiC preform. This enhances the interfacial property of SiC-matrix and improves the flexural strength of composites.

#### 3.1.2. Elastic Modulus

The measured elastic modulus of composites was reported in Table 2 as 174.2, 180.8, 195.7, 195.8, and 206.1 GPa for the 60.0%, 61.2%, 63.5%, 67.4%, and 68.0% composites, respectively. The effect of SiC volume fraction on the elastic modulus of composites is shown in Figure 2(b). It can be observed in Figure 2(b) that the elastic modulus of composites increases linearly with increasing SiC content, indicating that increasing the volume fraction of SiC particles can improve the elastic modulus of composites. When the SiC-matrix interfacial adhesion is strong, the stress transfer at SiC-matrix interface is efficient. The effect of SiC particle size on the elastic modulus of composites is shown in Figure 2(d). As depicted in Figure 2(d), for larger mean grain size of SiC particles (34.63–39.74 *μ*m), there is a prompt decrease in the elastic modulus with increasing particle sizes. For larger mean grain sizes of SiC particles 44.87 *μ*m, the increase in the flexural strength is no longer significant. Under effective stress transfer, the strength of composites will improve with the increase of SiC content, but the strain in the longitudinal and composites will decrease. So, the elastic modulus of composites increases with increasing SiC content.

#### 3.1.3. Poisson's Ratio

Poisson's ratios of composites are 0.36, 0.35, 0.35, 0.33, and 0.28 for the 60.0%, 61.2%, 63.5%, 67.4%, and 68.0% composites, respectively. The effect of SiC volume fraction on Poisson's ratio of composites is shown in Figure 2(e), in which Poisson's ratio of composites decreases with the increase of SiC volume fraction. The decrease of Poisson's ratio with the increase of SiC content is primarily due to the difference in property of SiC and matrix. The elastic modulus of SiC particle is greater than that of the matrix; and Poisson's ratio of SiC particles is lower than that of the matrix. The effect of SiC particle size on Poisson's ratio of composites is shown in Figure 2(f). As depicted in Figure 2(f), for larger mean grain size of SiC particles (34.63–39.74 *μ*m), there is a prompt increase in Poisson's ratio with increasing particle sizes. For larger mean grain sizes of SiC particles 44.87 *μ*m, the increase in Poisson's ratio is no longer significant. This resists the negative strain of the matrix in the transverse direction [16]. So, Poisson's ratio of composites with higher SiC content is less than Poisson's ratio of composites with lower SiC content.

#### 3.1.4. Fracture Behavior

**Figure 4:**
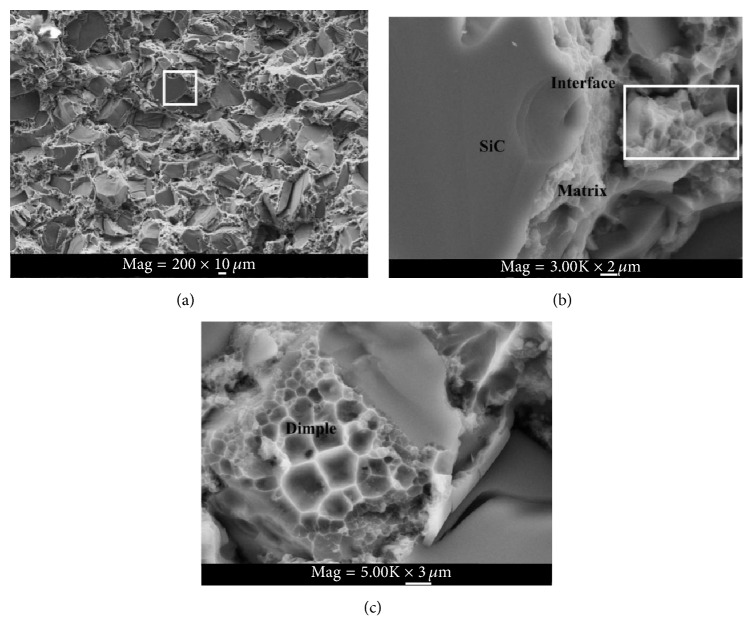
Fracture morphology of SiC_p_/Al composites. (a) The flexural fracture of the composite containing 68.0% SiCp, (b) high magnification image of the framed area in (a), and (c) high magnification image of the framed area in (b).

Figure 4(a) is the SEM fractograph for the fracture sample of 68.0% composites. The fracture surface of composites in Figure 4(a) is very rough. The fracture surface appearance of SiC particles presents variety, such as steps, crack, and mirror. The result indicates that the dominant fracture mechanism of composites is a cleavage fracture arising from crack propagation. The fracture of composites appears to have an obvious brittle character.  Figure 4(b) is highly magnified images of the framed area in Figure 4(a).  Figure 4(b) exhibits the interfacial adhesion between SiC particle and matrix is perfectly bonded. There is no debonding between the interface, SiC particle, and matrix in good fusion. The results reveal that the interfacial bonding strength between SiC particle and matrix is high [17].  Figure 4(c) is high magnification image of the framed area in Figure 4(b).  Figure 4(c) shows that there is an extensive dimple pattern in the local composite. It can be observed that the dimple sizes in the composite are less than 5 *μ*m. The dimples are associated with SiC particle size. Since there is incomplete infiltrating, the interfacial adhesion between fine particles and matrix is not perfect. Debonding will occur at the interface between fine particles and the matrix under the local stress concentrations. With ductile crack propagation, the void nucleation grows and finally coalesces. This results in dimple formation. Dimples on the composites show that ductile fracture occurs in the local composites during the breaking process of the composites. However, no dislocation is observed near crack tip in Figure 4(c). It can be concluded that the main character of composites behaves as brittle materials [18].

### 3.2. Properties of the Anodic Oxide Composites

#### 3.2.1. XRD Analysis of Nonoxidizable and Anodized SiCp/Al Composite

**Figure 5:**
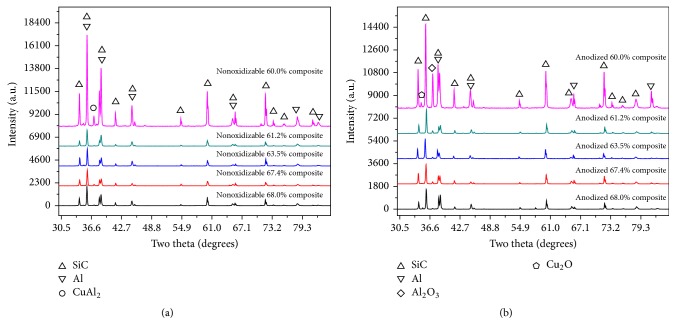
XRD patterns of the SiC_P_/Al composites (a) before and (b) after anodization.

Figure 5(a) displays the XRD results of the nonoxidizable composites with different volume fractions of SiC particles. In Figure 5(a), SiC, Al, and CuAl_2_ phases are identified by XRD; the Si and Mg peak are not observed, and the detrimental interfacial reaction producing Al_4_C_3_ also is not found. The contents of Si, Cu, and Mg listed in matrix (Table 1) are 0.760%, 2.085%, and 1.696%, respectively. However, the elements of Si and Mg cannot be detected in composites. Such result indicates that Si and Mg dissolve into the aluminum. The peak of CuAl_2_ shows that the chemical reaction between Al and Cu happens and that leads to generation of the new phase CuAl_2_. The composites without Al_4_C_3_ indicate that Si and Mg, though in much smaller amounts in the aluminum alloy, can effectively suppress the occurrence of the detrimental interface reaction: 4Al + 3SiC = Al_4_C_3_ + 3Si.

Figure 5(b) shows the XRD results of the anodized composites with different SiC content. In Figure 5(b), the peaks of SiC and Al are very strong, and the Al_2_O_3_ and Cu_2_O peaks are detected. The phase analysis reveals that oxide films formed on the surface of composites after anodic oxidation treatment. It is worth mentioning that the formation of Cu oxides is a suboxide. Petukhov et al. think that the formation of a suboxide is related to the mass transfer and the insufficient O^2-^ ions in electrolyte [19]. Chern and Tsai find that the suboxide on the surface of composites can improve the wettability of metal and obtain a higher binding force with glass [20]. So, the formation of suboxides in composites is helpful to compound glass and composites.

#### 3.2.2. Micrograph of Anodic Film

**Figure 6:**
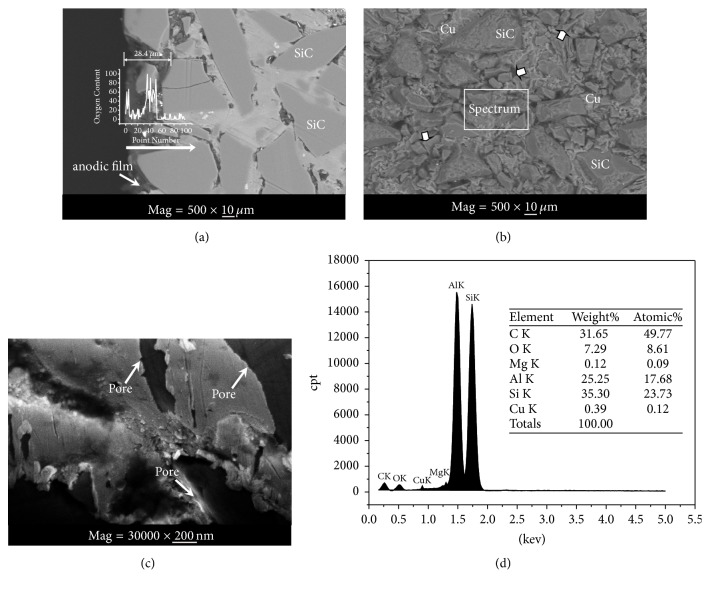
Micrograph of the Al 60.0 vol%  SiC_P_ composite after oxidation for 30 min: (a) backscattered electrons image of the cross section, (b) backscattered electrons image of the surface, (c) high magnification image of the anodized film, and (d) energy dispersive spectrum for the framed area in (b).

Figure 6(a) shows SEM micrograph of the anodized 60.0% SiCp/Al composite at room temperature for 30 min. For the image obtained by using electron backscattered diffraction (EBSD), the element oxygen appears darker than Si and Al because of its smaller atomic mass. In Figure 6(a), it can be observed that the surface of the composite is composed of two layers of materials: a bright layer and darker layer. The darker layer has penetrated into the composite and the thickness of darker layer can reach 28.4 *μ*m. The oxygen content at the interface between bright layer and darker layer is investigated by energy dispersive X-ray analysis (white arrow). The white curve in Figure 6(a) reflects the change of the oxygen between the bright layer and the darker layer. The result displays the oxygen content in the darker layer is obviously higher than in the bright layer. The phase analysis of composites after oxidation (Figure 5(b)) and the mutation of oxygen content are a strong argument for the saying that the dark layer on the surface of composite is the anodic film.

Figure 6(b) displays an EBSD image of the surface of the anodized 60.0% SiCp/Al composite. As seen from Figure 6(b), there are many darker regions (white arrow) on the surface of the composite, the distribution of these darker regions is uniform, and SiC particles are surrounded by the darker regions. An electron probe microanalysis (EPMA) is used to analyze the microscopic structure and composition of the composite's surface. The compositional elements of the framed area in Figure 6(b) are obtained by map analyses with EPMA. According to the composition determination, EPMA analysis shows that not only C, Cu, Al, Mg, and Si but also O exists on the composite. The result indicates that the surface of the composite is covered by oxide as shown in Figure 6(d).

Figure 6(c) shows high magnification morphology of anodic film. It can be obviously observed from Figure 6(c) that the microscopic structure of anodic film is a porous structure. The shape of the pore looks like a crooked pipe. The shape of the pore is related to the shape of the matrix. The tightly packed SiC particles determine that the structure of matrix is complicated, so the channel of mass transfer results in the crooked pore.

#### 3.2.3. Film Thickness

The thickness of oxidation layers under different anodizing time is presented in Table 4.

**Figure 7:**
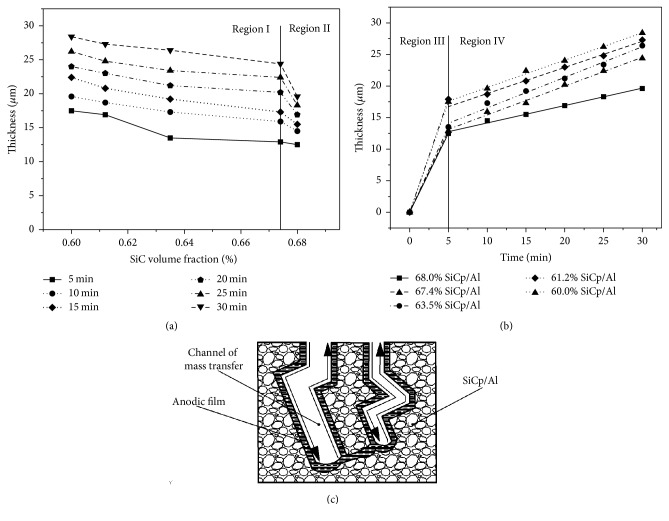
The relationship (a) between the anodic film thickness and SiC volume fraction and (b) between the anodic film thickness and anodizing time. (c) The schematic of mass transfer channel.

**Figure 8:**
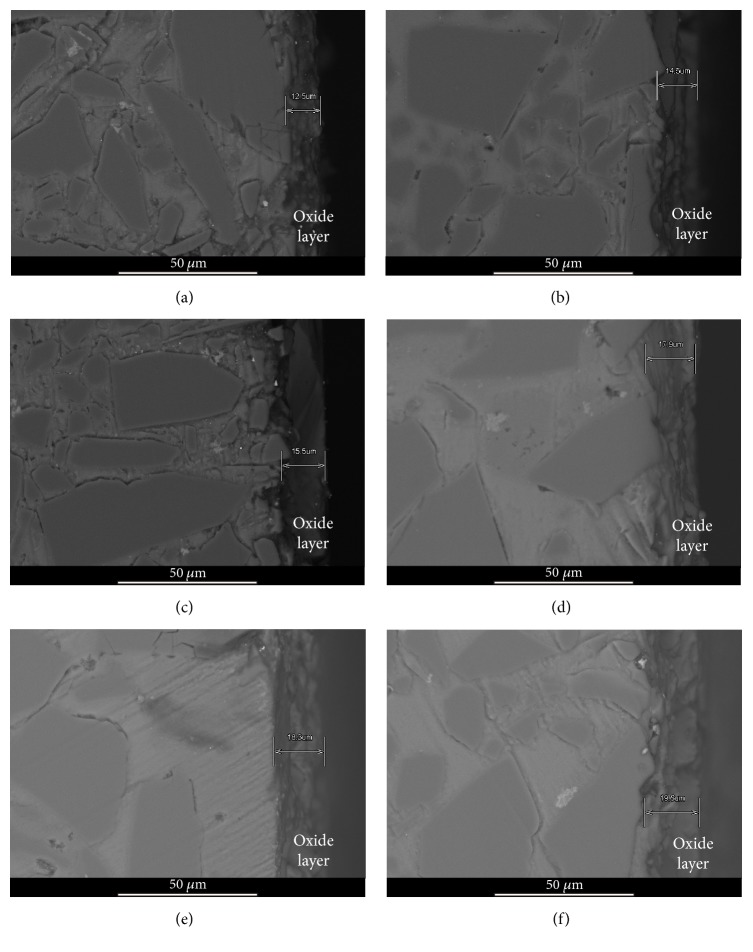
SEM micrograph of 68.0% SiCp/Al composite after being anodized with different time.

The result is in agreement with the reports about the growth of oxide film [21, 22]. The thickness of the anodic films obtained during this experiment ranges from 12.5 to 28.4 *μ*m. Similarly, the thickness of the anodic films in the linearity ranges from 5 to 76 *μ*m [23].  Figure 7(a) displays the relationship between the film thickness and SiC volume fraction. It can be observed that the thickness of oxidation layers decreases with the increase of SiC volume fraction for the same anodizing time, and the decline rate in region I is larger than that in region II. The result implies that the SiC contents in composites have an effect on the growth rates of anodic films. The more the SiC particles content, the higher the packing density of SiC particle, and the more complex the structure of matrix; so it was the structure of the porous anodic oxide film. The structure of mass transfer channel is illustrated in Figure 7(c). During anodic oxidation of composites, the growing anodic films need a continuous delivery of oxygen [24]. The growth of the anodic films is determined by the transfer velocity of ion across the matrix-oxide interface [25]. Because of the complex structure, the mass transfer channel obstructs the flow of electrolytes, and this results in a reduction of the mas transfer velocity and the decline in the growth rates of anodic films. The 2 *μ*m SiC particle content of 68% composite is more than that of 67.4% of the composite. The increase of 2 *μ*m SiC particle accelerates complication of the mass transfer channel's structure. So, the flow resistance of electrolytes increases sharply. As a result, the growth rate of anodic films decreases significantly. The relationship between the thickness of anodic films and time is shown in Figure 7(b). From Figure 7(b), it is clear that film growth is consistent with the rising stage (region III) and the stable stage (region IV). The film growth rate of the rising stage is greater than that of the stable stage. At the same time, the film thickness-time relationship reveals that the anodic films grow linearly with time during the stable stage. The result implies that there is dynamic equilibrium between oxidation and chemical dissolution. The dynamic equilibrium of the oxide formation and dissolution causes that the growth rates of anodic films are a constant. The thickness of SiCp/Al content 68.0% micrograph was shown in Figure 8.

#### 3.2.4. The Melting Point of Composites

**Figure 9:**
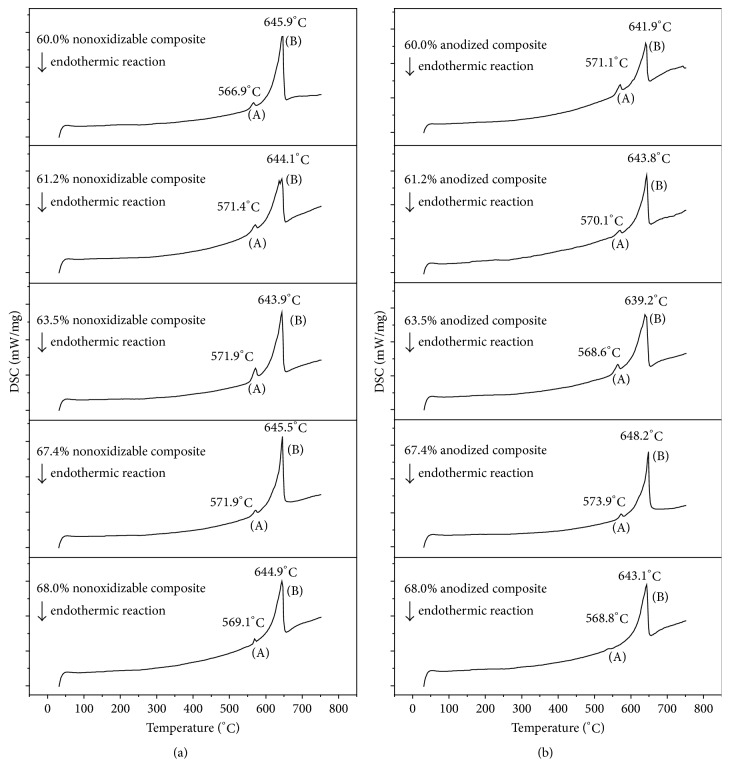
DSC curves of the composite containing 60.0 vol% SiCp, (a) before and (b) after anodization.

The DSC thermograms of nonoxidizable and anodized composites obtained are shown in Figures 9(a) and 9(b), respectively. It is found that the curves in both Figures consist of similar principle features and heat effects are the two endothermic peaks, (A) and (B). The endothermic peak (A) is due to the dissolution of CuAl_2_, and the endothermic peak (B) is attributed to the dissolution of the matrix alloy [26]. The peak temperatures (A and B) are shown in Figures 9(a) and 9(b). The data in Figures 9(a) and 9(b) show the following: (1) the endothermic peaks (A) of all nonoxidizable composites and anodized composites are near 570°C, and the endothermic peaks (B) of all nonoxidizable composites and anodized composites are near 640°C. During compounding optical glass and composites, the preheat temperature must be based on peak (A) temperature. (2) SiC content and anodic films on the surface of composites have a negligent effect on the endothermic peaks of composites.

## 4. Discussions

At present, a number of scholars have applied micromechanics theory to study the quantitative relationship between the properties of composites and the properties of its constituents, and established the mathematical model, such as the Hashin-Shtrikman model [27] (H-S model) and the Wu model [28]. By comparing the predicted results with the model with experimental results, the rationality of the model can be studied. However, the accuracy of these models on predicting the elastic modulus of high volume fraction SiC_P_/Al composites is rare in reports, let alone model validation. The advance of the present composite is the fabrication process, which consisted of mixing SiC particles, sintering process design, prefrom quality traceability, preheating prefrom, melting aluminum, and squeeze infiltrating. The new process differs from the traditional process, which can decrease the rejection rate and cost. The development of SiCp/Al composite is integration of material and process development with system design and manufacturing process, which provides an approach to obtain the maximum benefit from the characteristics offered by a new material [29].

### 4.1. Hashin-Shtrikman Model

Hashin and Shtrikman consider the strain, interface bonding, and stress transfer between reinforcement and matrix. They assume that the strain cross section of the composite under uniaxial loading is uniform, the interface bonding between particle-matrix is perfect, and stress transfer between reinforcement and matrix is effective. They have grouped many analysis methods, such as direct methods, variation methods, and approximation methods. Finally, they derived a model for the effective elastic modulus of two-phase composite materials. The model is expressed as follows:

(1) $$\begin{array}{c} E_{c}=E_{m}\frac{\left[E_{m}V_{m}+E_{p}\left(V_{p}+1\right)\right]}{\left[E_{p}V_{m}+E_{m}\left(V_{p}+1\right)\right]}, \end{array}$$

where *E* is the elastic modulus and *V* is the volume fraction. Subscripts *c*, *m*, and *p* refer to the composite, matrix, and particle, respectively.

The predicted results of the H-S model are presented in Table 2. Figure 2(b) shows the comparison between the prediction and experimental data. As can be seen, the measured elastic moduli are lower than the predicted value of H-S model but can reach 70% of them. The result indicates that if the modulus of high volume fraction SiC_P_/Al composite is calculated by H-S model, the accuracy of predicted value is low. The reason can be attributed to the perfect hypothesis on the interface between reinforcement and matrix. The dimples shown in Figure 4(c) indicate that the local composites have ductile characteristic. The ductile characteristic will increase the strain of composites. Decohesion between the SiC and matrix will cause an ineffective stress transfer between SiC and matrix and lead to a decline in the strength of the composites. These will result in the decrease of the moduli.

### 4.2. Wu Model

In consideration of the difficulties for characterizing the microstructures of two-phase composites, Wu introduces an unknown parameter, which can be determined from experiment. He derives an equation for calculating the effective elastic modulus of composites. Wu's equation can be written as follows:

(2) $$\begin{array}{c} \frac{1}{E_{c}}=\left[\;\frac{1}{E_{m}}-\frac{{\left(1/E_{m}-1/E_{p}\right)}^{2}}{\lambda \left(1/E_{m}-V_{m}/V_{p}E_{m}\right)}\;\right]V_{m}+\frac{V_{p}}{E_{p}}, \end{array}$$

where *E* is the elastic modulus, *V* is volume fraction, *λ* is the unknown parameter (*λ* = 20), and subscripts *c*, *m*, and *p* refer to the composite, matrix, and particle, respectively.

The calculated data from the Wu model are listed in Table 2. By contrasting measured data with forecasting value, it can be found that the Wu model values are also higher than the measured moduli. However, the measured elastic modulus can reach 78% the prediction of the Wu model, indicating that Wu model is more practical for the prediction of high volume fraction composites' modulus. It is due to the unknown parameter (*λ*) that can fit the experimental data.

## 5. Conclusions

In the present study, the mechanical and anodized surface properties of high volume fraction SiC_P_/Al composite have been investigated, and the accuracy of theoretical model in predicting the elastic modulus of high volume fraction composites also has been verified. The following results are obtained.

Si and Mg are added in smaller amounts to the aluminum alloy, effectively suppressing the formation of Al_4_C_3_. With an increase in SiC volume fraction, the flexural strength and Poisson's ratio decrease while the elastic modulus increases. With the mean grain size increasing, the flexural strength increases. From the fracture feature, it is found that the dominant fracture mechanism of composites is a cleavage fracture with occurrence of ductile fracture in the local of composites.

Through anodic oxidation treatment, an oxidation film with porous structure can be prepared on the surface of the composites. The anodic film is uniformly distributed. The oxide growth rate of composites linearly increases with anodizing time and decreases with SiC content increasing. SiC content and anodic films on the surface of composites have a negligent effect on the endothermic peaks of the composites.

The measured elastic modulus is in good agreement with predicted values based on Wu's model.

## Figures and Tables

**Table 1:** Chemical composition of 6061-Al.

Specification	Composition (wt %)							
	Cu	Mg	Fe	Si	Zn	Mn	Pb	Al
Aluminum alloy	0.258	1.08	0.255	0.962	0.24	0.168	0.09	96.947

**Table 2:** The properties of the prepared SiCp/Al composites. *D* is the average diameter of the mixed powders used in this work, *V*_*p*_ is the volume fraction of SiC particles, *σ*_*bb*_ is the flexural strength of the composites, *E* is the elastic modulus of the composites, and *γ* is Poisson's ratio of the composites.

Gradation composition (*µ*m) and proportion	*D* *μ*m	*V*_*p*_ (%)	*σ*_*bb*_ (MPa)	*γ*	*E* (GPa)			Measured/Wu (%)	Measured/H-S (%)
					Measured data	Wu model	H-S model		
45 : 8 : 2 = 1000 : 100 : 400	34.63	68.0	483	0.28	206.1	245.1	271.7	84	76
45 : 8 : 2 = 1000 : 200 : 300	36.69	67.4	544	0.33	195.8	243.3	269.8	80	73
45 : 8 : 2 = 1000 : 250 : 250	37.38	63.5	548	0.35	195.7	231.9	257.7	84	76
45 : 8 : 2 = 1000 : 300 : 200	39.74	61.2	554	0.35	180.8	225.6	251.0	80	72
45 : 8 : 2 = 1000 : 400 : 100	44.87	60.0	569	0.36	174.2	222.5	247.5	78	70

**Table 3:** The properties of SiC particles and aluminum alloy at room temperature. *σ*_*bb*_, *E*, and *γ* represent flexural strength, elastic modulus, and Poisson's ratio, respectively.

Specification	*σ*_*bb*_	*E*	*γ*
	(MPa)	(GPa)	
SiC	550	410	0.14
Aluminum alloy	398^a^	130.1^a^	0.40^a^

^a^Experimental data.

**Table 4:** The thickness of anodized film after oxidizing for different time. *V*_*p*_ is the volume fraction of SiC particles.

*V*_*p*_	Thickness of anodized film for different oxidizing time (*μ*m)					
(%)	5 min	10 min	15 min	20 min	25 min	30 min
68.0	12.5	14.5	15.5	17.9	18.3	19.6
67.4	12.9	15.9	17.3	20.2	22.4	24.4
63.5	13.5	16.3	19.2	21.2	23.4	26.4
61.2	16.9	18.7	20.8	23.0	24.8	27.3
60.0	17.5	19.6	22.4	24.0	26.2	28.4
